# Effects of Forage-to-Concentrate Ratio During Cold-Season Supplementation on Growth Performance, Serum Biochemistry, Hormones, and Antioxidant Capacity in Yak Calves on the Qinghai–Tibet Plateau

**DOI:** 10.3390/ani15172490

**Published:** 2025-08-25

**Authors:** Yuhong Bao, Jia Zhou, Xuetao Yang, Ruizhi Shi, Yangci Liao

**Affiliations:** 1 Tibet Academy of Agricultural and Animal Husbandry Sciences, Lhasa 850000, China; yangxt@taaas.org (X.Y.); r231119569@163.com (R.S.); lyangci@taaas.org (Y.L.); 2 Chongqing Academy of Animal Sciences, Chongqing 402460, China; zhoujia1@stu.sicau.edu.cn

**Keywords:** yak calves, forage-to-concentrate, cold-season supplementation, growth performance, serum biochemistry, hormone profile, antioxidant capacity

## Abstract

This study investigated the effects of different forage-to-concentrate (F:C) ratios during cold-season supplementation on growth performance, serum biochemical parameters, hormone levels, and antioxidant capacity in yak calves on the Qinghai–Tibet Plateau. Eighteen 8-months-old male yaks with similar body weights (110.01 ± 2.08 kg) were randomly assigned to two groups receiving diets with high (F:C = 7:3) or low (F:C = 3:7) forage. The trial lasted 60 days, including early (days 0–30) and late (days 31–60) experimental stages. Body weight was measured, and serum samples were collected on days 30 and 60 for biochemical and hormonal analyses. Yaks in the low-forage group showed significantly greater average daily gain during both stages and the entire experiment (*p* < 0.05), with a 7.92% increase in final body weight. Serum total protein and globulin levels were significantly higher in the low-forage group throughout the trial (*p* < 0.05 or *p* < 0.01), while other biochemical parameters remained unaffected. Growth hormone concentrations were significantly elevated in the low-forage group at both stages (*p* < 0.05). Additionally, malondialdehyde levels tended to decrease in the early stage (*p* = 0.056), and total antioxidant capacity was significantly lower in the late experimental stage (*p* = 0.040) in the low-forage group. A higher net economic benefit was observed in the group fed an F:C ratio of 3:7 than in the 7:3 group. These findings suggest that cold-season supplementation with a low-forage diet improves growth performance and protein utilization in yak calves but may negatively impact antioxidant status. An optimal F:C balance should therefore be considered to support both performance and health in yak husbandry under harsh environmental conditions.

## Simple Summary

Yaks are vital to the livelihoods of herders on the Qinghai–Tibet Plateau, yet they often suffer from poor nutrition during the long, harsh cold season, leading to significant weight loss and reduced productivity. This study examined how different forage-to-concentrate (F:C) ratios in winter supplements affect growth performance, blood biochemistry, hormone levels, and antioxidant status in young yaks. The results showed that diets with a higher concentrate ratio (F:C = 3:7) significantly improved growth performance and protein utilization, likely due to elevated growth hormone levels. Moreover, this dietary strategy contributes to increased net economic returns for herders. However, this diet also reduced the animals’ antioxidant capacity, suggesting a potential health trade-off. These findings highlight the importance of optimizing the F:C ratio in cold-season feeding strategies to ensure both better growth and overall health in yak husbandry.

## 1. Introduction

Yaks (*Bos grunniens*) play a crucial role in the pastoral economy of the Qinghai–Tibet Plateau, supplying essential resources such as milk, meat, wool, and fuel, and serving as a means of transportation [1]. As the only bovine species capable of surviving and reproducing at elevations above 3000 m, yaks are indispensable to local livelihoods [2]. However, traditional grazing systems have become increasingly inadequate, particularly during the prolonged cold season characterized by limited forage availability, resulting in nutritional deficiencies, compromised health, and suboptimal growth performance [3]. Moreover, the rising yak population and ongoing grassland degradation have diminished the carrying capacity of natural pastures, thereby restricting the supply of high-quality meat and impeding the sustainable development of the yak meat industry [4]. Currently, yak husbandry remains largely dependent on traditional free-range grazing. Due to the region’s harsh climatic conditions, yaks are subjected to an extended cold season lasting from October to May. During this period, the scarcity of high-quality forage leads to severe energy and nutrient deficits. As a result, adult yaks may lose up to 25% or more of their body weight [5,6]. This cycle—characterized by weight gain in summer and autumn, followed by significant weight loss in winter and increased mortality in spring—is commonly described as “fat in summer and autumn, thin in winter, and dead in spring.”

Nutritional deficiencies during the cold season have been shown to adversely affect the physiological status of grazing yaks, particularly protein metabolism [7,8,9]. Recent studies have demonstrated that nutritional regulation through concentrate supplementation during the cold season can effectively improve the health and performance of grazing yaks [8,9,10]. However, modern intensive feeding systems—characterized by high-concentrate diets—tend to promote greater daily weight gain in yaks. This approach, however, neglects the unique characteristics of the yak rumen microbiota, which exhibits a superior fiber-degrading capacity [11]. Diets dominated by concentrates often violate the recommended 40–70% roughage intake typical for ruminants, leading to the excessive accumulation of volatile fatty acids in the rumen. This disrupts fermentation balance and allows fermentable carbohydrates to reach the intestine, potentially resulting in acidosis and microbial dysbiosis [12].

Therefore, an appropriate dietary structure during cold-season supplementation is essential. A balanced ratio of roughage and concentrate not only supports growth and development in ruminants but also enhances the digestion, absorption, and utilization of various nutrients, closely linked to improved metabolic function [13,14]. Despite this, limited research has investigated the metabolic responses of yaks to different forage-to-concentrate (F:C) ratios during cold-season feeding. Previous studies have indicated that increasing the proportion of concentrate in the diet (decreasing the F:C ratio from 7:3 to 1:9) can result in a secondary improvement in average daily gain (ADG) and slaughter rate in fattening sheep, with an optimal effect observed at an F:C ratio of 3:7 [15]. Similarly, in Angus cattle, decreasing the F:C ratio from 65:35 to 35:65 has been reported to enhance ADG, improve rumen fermentation, increase ammonia nitrogen production, and elevate the concentrations of acetate and propionate, while reducing the acetate-to-propionate ratio. However, this was accompanied by a significant decrease in ruminal pH [16]. Notably, an F:C ratio of 2:8 has been shown to alter rumen fermentation patterns, lower rumen pH, and negatively impact rumen health in yaks [14]. Notably, an F:C ratio of 2:8 has been shown to alter rumen fermentation patterns, reduce rumen pH, and negatively affect rumen health in yaks [14]. Therefore, based on previous studies and to avoid compromising yak health, a diet with an F:C ratio of 7:3 was assigned to the high-forage group, while the low-forage group was fed a diet with an F:C ratio of 3:7 in this study as an initial exploration, aiming to provide a basis for supplementary feeding strategies in yaks. Furthermore, the growth pattern of yaks follows an exponential trajectory, marked by a rapid early growth phase followed by a deceleration in growth rate. The period between 6 and 12 months of age is considered a critical window for growth intervention in yaks [17]. An appropriate dietary nutrient level is essential for promoting growth in ruminants. Excessive concentrate levels may suppress growth, lead to feed waste, and increase production costs, whereas insufficient concentrate proportions may limit growth performance. Therefore, providing an optimal level of concentrate supplementation during the growing phase is particularly important. It was therefore hypothesized that increasing the dietary concentrate proportion could improve nutrient composition, thereby enhancing protein metabolism and promoting growth in yaks; however, potential health risks may arise compared to a low-concentrate diet. Determining an optimal F:C ratio is critical for promoting healthy growth in yaks during the cold season. In this study, the effects of high- and low-forage supplementation on growth performance in growing yaks were compared. Additionally, serum biochemical parameters, hormone levels, and antioxidant indices were assessed to provide a scientific basis for the healthy and efficient development of yak husbandry.

## 2. Materials and Methods

This study was conducted in accordance with the Chinese Animal Welfare Guidelines, and all experimental procedures were approved by the Animal Care and Ethics Committee of the Tibet Academy of Agriculture and Animal Husbandry Sciences (Approval No. TAAAHS-2023–4).

### 2.1. Animals and Experimental Design

The experiment was conducted at the Nyangya Yak Breeding Industry Development Co., Ltd., located in Jiali County, Nagqu City, Tibet Autonomous Region (N36°35′, E102°46′). The site is situated in southeastern Nagqu, between the Tanggula and Nyainqêntanglha mountain ranges, at an average elevation exceeding 4500 m. The region is characterized by a plateau subalpine semi-humid monsoon climate, with annual precipitation ranging from 500 to 700 mm and a mean annual temperature of −0.9 °C. The climate is dry, cold, and oxygen-deficient. The pasturelands are primarily alpine meadows dominated by *Kobresia pygmaea* and *Kobresia tibetica*. The growing season for forage grasses typically begins in late May and lasts for approximately 102 days, while biomass production during the cold season is minimal. The study was carried out between December 2023 and January 2024. During this period, the average maximum and minimum temperatures were 3 °C and −17 °C, respectively.

A total of 18 healthy male yak calves (8 months old, 110.01 ± 2.08 kg) were randomly assigned to two dietary treatment groups (*n* = 9 per group), receiving supplemental diets with different forage-to-concentrate (F:C) ratios. The high-forage group was fed a diet with an F:C ratio of 7:3, while the low-forage group received a diet with an F:C ratio of 3:7. The experimental diets were formulated to reflect practical feeding practices commonly used in the region. Owing to the substantial variation in forage-to-concentrate ratios and consistent with challenges reported in previous studies [15,18], it was not possible to formulate iso-nitrogenous diets across treatments. However, particular attention was given to maintaining comparable levels of key mineral nutrients, especially calcium and phosphorus, to minimize potential confounding effects.

All yaks were transferred to a shared grazing pasture at 07:30 each morning and returned to their pens at 19:30, where they were individually supplemented with an experimental diet equivalent to 1% of their body weight. The composition of the diets is shown in Table 1. The nutrient composition of the total mixed ration, in which concentrates and forages were thoroughly blended, was determined in accordance with methods established in our previous research [6]. Briefly, DM was determined according to AOAC (2005; methods 930.15) [19]. Nitrogen content was analyzed using the Kjeldahl method (AOAC, 2005; method 981.10) [19] and converted to crude protein (CP) as N × 6.25. Ash content was determined by complete combustion in a muffle furnace at 600 °C for 6 h (AOAC, 2005; method 942.05) [19]. Calcium and phosphorus were analyzed by flame atomic absorption spectrometry and fluorescence spectrophotometry, respectively (AOAC, 1990; methods 985.35 and 986.24) [20]. Neutral detergent fiber (NDF) and acid detergent fiber (ADF) were determined according to Van Soest et al. (1991) [21]. Fresh and clean water was available ad libitum throughout the trial. The experimental period lasted 60 days, with days 0–30 defined as the early experimental stage and days 31–60 as the late experimental stage.

### 2.2. Growth Performance

All yaks were weighed individually in the morning on days 0, 30, and 60 after an overnight fast. Average daily gain (ADG) was calculated for the early (days 0–30), late (days 31–60), and overall (days 0–60) periods using the following formula:

ADG = (Final body weight − Initial body weight)/Number of days.

### 2.3. Sample Collection

Blood samples were collected from all yaks on days 30 and 60 in the morning. Approximately 3 tubes of 5 mL of blood were drawn from the jugular vein using sterile needles and collected into non-anticoagulant tubes. The samples were left to stand at room temperature for 1 h, then centrifuged at 3000 rpm for 15 min. The resulting serum was aliquoted into 1 mL cryovials and stored at −20 °C until further analysis.

### 2.4. Biochemical Analysis

Serum biochemical parameters, including total protein (TP), albumin (ALB), glucose (GLU), blood urea nitrogen (BUN), total cholesterol (TC), triglycerides (TG), free fatty acids (FFA), low-density lipoprotein cholesterol (LDL-C), and high-density lipoprotein cholesterol (HDL-C), were quantified using colorimetric methods with commercial assay kits (Meikang Biotechnology Co., Ltd., Zhoushan, Zhejiang, China). Analyses were performed with an automated biochemical analyzer (Model 7020, Hitachi, Tokyo, Japan). Globulin (GLO) level was derived mathematically by subtracting ALB from TP values.

Serum concentrations of metabolic hormones, including thyroxine (T4), triiodothyronine (T3), growth hormone (GH), leptin (LEP), insulin (INS), and insulin-like growth factor-1 (IGF-1), were measured using commercial ELISA kits (Jiangsu Jiancheng Bioengineering Institute, Beijing, China) according to the manufacturer’s instructions.

Antioxidant indicators—including superoxide dismutase (SOD), malondialdehyde (MDA), catalase (CAT), total antioxidant capacity (T-AOC), and glutathione peroxidase (GSH-Px)—were measured in 100 μL serum samples using commercial assay kits according to the manufacturer’s instructions (Nanjing Jiancheng Bioengineering Institute, Nanjing, China).

### 2.5. Economic Benefit

Breeding profit was calculated by the difference between the benefit of live-weight gain and total feed cost. Net economic benefit (NEB) was determined by the following equation [24]:

NEB = (*G_w_* × *P_m_*)/(*T_e_* × *DMI* × *P_d_*) − 1

where *G_w_* is the total live-weight gain (kg), *P_m_* is the market unit price of live animals (CNY/kg), *T_e_* is the experiment time (day), *DMI* is the daily DM intake (kg/d), and *P_d_* is the unit price of the given diet (CNY/kg).

### 2.6. Statistical Analysis

The experimental data were analyzed using one-way analyses of variance (ANOVA) followed by Duncan’s multiple comparisons in SPSS 26.0 Statistics software (SPSS Inc., Chicago, IL, USA):

*Y_ij_* = *μ* + *α_i_* + *e_ij_*,

where *Y_ij_* is the dependent variable; *μ* is the overall mean; *α_i_* is the effect of the *i*-th group; and *e_ij_* is the random error. Data are presented as mean ± standard error of mean (SEM). Differences were considered statistically significant at *p* < 0.05, and a tendency toward significance was defined as 0.05 ≤ *p* ≤ 0.1.

## 3. Results

### 3.1. Effects of Forage-to-Concentrate Ratio on Growth Performance and Economic Return in Growing Yaks

As shown in Table 2, there was no significant difference in initial body weight between the two groups on day 0 (*p* = 0.857). However, by day 60, the low-forage group exhibited a trend toward increased body weight compared to the high-forage group (*p* = 0.072), with an improvement of 7.92%. The ADG during the early, late, and entire experimental periods was significantly higher in the low-forage group than in the high-forage group (*p* < 0.05). Breeding profit and NEB during the cold season were increased for local herders when yaks were fed with low-forage diets compared to high-forage diets.

### 3.2. Effects of Forage-to-Concentrate Ratio on Serum Biochemical Parameters in Growing Yaks

The effects of dietary forage-to-concentrate ratio on serum biochemical indices are presented in Table 3. During the early stage, TP and GLO concentrations were significantly higher in the low-forage group compared to the high-forage group (*p* < 0.05). In the late stage, these differences became more pronounced, with both TP and GLO levels showing highly significant increases (*p* < 0.01). No significant differences were observed between groups in ALB, GLU, BUN, TC, TG, FFA, LDL-C, or HDL-C concentrations (*p* > 0.05).

### 3.3. Effects of Forage-to-Concentrate Ratio on Serum Hormones in Growing Yaks

As shown in Table 4, supplementation with a low-forage diet significantly increased serum GH concentrations in both early and late stages compared to the high-forage group (*p* < 0.05).

### 3.4. Effects of Forage-to-Concentrate Ratio on Serum Antioxidant Capacity in Growing Yaks

According to Table 5, during the early stage, a decreasing trend in MDA concentration was observed in the low-forage group (*p* = 0.056). In the late stage, T-AOC was significantly lower in the low-forage group compared to the high-forage group (*p* = 0.040).

## 4. Discussion

The Qinghai–Tibet Plateau is characterized by distinct seasonal temperature fluctuations. During the warm season, pasture yield and nutritional quality are high, allowing grazing yaks to meet their forage intake requirements by traveling only 3–5 km per day [27]. The abundance of forage leads to rumen fill, reducing concentrate intake. In contrast, the cold season is marked by withered, nutrient-deficient forage, and grazing alone fails to meet the yak’s requirements for maintenance and growth. Consequently, yaks exhibit hunger-driven foraging behavior, extending their daily range to approximately 10 km [27]. Less than half of the energy consumed during the warm season is available for consumption in the winter [5]. Concentrate supplementation during the cold season can promote compensatory growth and significantly improve yak performance. However, in practice, supplementation often involves direct provision of concentrates such as corn, soybean meal, rapeseed cake, wheat bran, or urea–molasses mixtures, which may pose risks to rumen health. Therefore, providing an optimal level of concentrate supplementation during the growing phase is particularly important. Nevertheless, there remains a lack of research on dietary F:C during supplementation. This study was designed to compare high and low-forage diets during the cold season, evaluating their effects on growth performance, serum biochemical profiles, hormone levels, and antioxidant capacity in growing yaks, with the goal of informing evidence-based supplementation strategies.

Previous research indicates that during the cold season, nutritional deficiencies exert a greater influence on the physiological status of grazing yaks than cold stress. Significant alterations in serum metabolic profiles, particularly those related to protein and amino acid metabolism, have been observed under nutritional stress. In fact, the effects of nutrient deficiency on protein and amino acid metabolism are more pronounced than those on energy or mineral metabolism during cold periods [7]. Studies have shown that cold-season supplementation with 0.8 kg/day of barley, rapeseed meal, or a combination of both can increase ADG in yaks by 38.5% to 61.5% [10]. Similarly, supplementation with barley or rapeseed meal during the warm season has been reported to enhance ADG by 97.1% to 121% [3]. Other findings suggest that daily supplementation with 0.5 kg of concentrate can maintain body weight, while 2.5–4.5 kg of concentrate can significantly improve ADG in yaks during winter [28]. These findings align with the present study, where low-forage supplementation resulted in a 57.32% increase in ADG compared to the high-forage group. Related research also supports the conclusion that high-energy or high-protein diets in winter enhance yak growth performance [9,29]. It is hypothesized that increasing the forage-to-concentrate ratio enhances digestive efficiency by accelerating digesta passage, providing more substrates for microbial proliferation and fermentation, and thereby improving nutrient absorption and utilization in yaks.

Complex physiological and biochemical processes occur continuously within animal organisms. Dietary components directly influence these processes by stimulating glucose uptake in skeletal muscle, heart, liver, and adipose tissues; modulating glucose and fatty acid oxidation; and regulating the synthesis of glucose, glycogen, and lipids in the liver and adipose tissue, as well as lipolysis in adipocytes [30]. Yaks possess unique regulatory mechanisms that enable them to withstand cold-season stress. During winter, increased fibrolytic enzyme activity and a higher relative abundance of fiber-degrading and VFA-producing microbes have been observed. Additionally, genes involved in VFA synthesis, absorption, and transport are significantly enriched in the rumen epithelium. The upregulation of genes related to VFA metabolism and arginine biosynthesis is believed to enhance energy compensation and promote efficient nitrogen utilization under the extreme environmental conditions of the Qinghai–Tibet Plateau [31]. Relevant studies have shown that increasing the dietary metabolizable energy from 7.20 to 8.58 MJ/kg DM during the cold season elevates serum glucose levels and total VFA concentrations in rumen fermentation, while also improving amino acid metabolism and increasing rumen microbial protein concentration [9]. Serum biochemical parameters are commonly utilized to evaluate the physiological and metabolic status of animals, providing insights into their health status [32]. In this study, serum TP and GLO concentrations were significantly higher in the low-forage group compared to the high-forage group. This finding is consistent with previous studies reporting that yaks grazing in October exhibited significantly higher serum TP and GLO levels than those during the cold season (December to March) [7]. Similarly, Zhou et al. [10] demonstrated that winter supplementation with concentrate mixtures positively affected plasma TP and albumin levels. Serum TP is considered a direct indicator of protein digestion, absorption, and overall dietary protein status. Higher TP concentrations generally reflect better protein quality in the diet, while severe protein malnutrition is typically associated with reduced TP levels. The observed increase in TP may be related to elevated concentrations of ruminal ammonia nitrogen and microbial crude protein, which are often diminished by prolonged fasting [7,33,34]. In addition, in this study, the low-forage diet contained a higher crude protein concentration compared to the high-forage diet. This difference in dietary protein levels is likely a significant contributor to the enhanced protein metabolism observed in yaks fed the low-forage diet. Furthermore, previous studies have reported that increasing the proportion of concentrate in the diet leads to an increase in the relative abundance of starch-degrading genera such as *Ruminococcus*, *Atopobium*, and unclassified *Clostridiales*, while the relative abundance of fiber-degrading bacteria is decreased. This microbial shift has been shown to enhance ruminal nitrogen metabolism, elevate ruminal ammonia nitrogen concentration, and improve protein utilization [16,35]. This is consistent with the observed increase in GH levels. The GH/IGF-1 axis plays a crucial role in regulating growth, maintaining bone mass, and promoting cell differentiation. Under favorable nutritional conditions, GH, in the presence of insulin, can promote amino acid uptake and enhance RNA and protein synthesis [36]. These results suggest that diets with increased concentrate levels can enhance protein utilization in yaks.

Animals possess enzymatic systems that counter oxidative stress, primarily consisting of SOD, CAT, and GSH-Px [37]. When reactive ROS are generated, SOD catalyzes their conversion into hydrogen peroxide (H_2_O_2_) and other peroxides, while CAT and GSH-Px facilitate the decomposition of H_2_O_2_. GSH-Px also catalyzes the conversion of lipid peroxides into alcohols. MDA, a product of unstable lipid peroxides, can cause cellular damage. T-AOC reflects the combined activity of antioxidants and antioxidant enzymes, serving as a comprehensive indicator of antioxidant function. In this study, serum MDA concentrations showed a decreasing trend in the low-forage group during the early stage of supplementation. However, in the later stage, T-AOC was significantly lower in the low-forage group compared to the high-forage group. These results indicate that the structure of the supplemental diet should be carefully considered during cold-season feeding, as prolonged high-concentrate supplementation may impair the antioxidant capacity of yaks.

## 5. Conclusions

This study investigated the effects of different forage-to-concentrate (F:C) ratios on the growth, serum biochemical parameters, hormones, and antioxidant capacity of yak calves during the cold season. Results showed that supplementation with a low-forage diet (F:C ratio of 3:7) significantly improved growth performance, with higher average daily gain and body weight gain compared to the high-forage group (F:C ratio of 7:3). Additionally, serum total protein and globulin levels were higher in the low-forage group, suggesting enhanced protein utilization. However, the low-forage group also showed a reduced total antioxidant capacity in the later phase of the experiment, indicating potential oxidative stress. In conclusion, while a low-forage diet supports better growth performance in yaks, enhances protein utilization, and increases the net economic benefit for herders, careful consideration of its potential impacts on rumen health and oxidative stress is necessary. This study offers preliminary insights into appropriate F:C ratios for the supplementary feeding of yak calves during the cold season. Further research is warranted to more comprehensively examine F:C ratios ranging from 7:3 to 3:7, with the aim of supporting their healthy growth.

## Figures and Tables

**Table 1:** Ingredient and nutrient levels of diets (%, DM basis, unless otherwise stated).

Ingredients	High Forage	Low Forage
Corn	21.19	52.21
Soybean meal	6.56	15.54
Limestone powder	0.50	0.50
Dicalcium phosphate	0.25	0.25
Sodium chloride	0.50	0.50
Vitamin premix ^1^	0.50	0.50
Mineral premix ^2^	0.50	0.50
Oat hay	70.00	30.00
Total	100.00	100.00
Nutrient levels ^3^		
NEmf, MJ/kg	4.39	6.30
CP, %	10.50	13.68
EE, %	2.34	3.15
NDF, %	47.17	28.24
ADF, %	30.90	17.33
Ca, %	0.43	0.39
P, %	0.38	0.31
NFC, %	37.79	52.83
NFC/ADF	0.80	1.88

NEmf = net energy for maintenance and fattening; CP = crude protein; EE = Ether extract; NDF = neutral detergent fiber; ADF = acid detergent fiber; Ca = calcium; P = phosphorus; NFC = Non-fiber carbohydrates. ^1^ The premix provides per kg of diet: Vitamin A 8800 IU, Vitamin D 1600 IU, Vitamin E 360 IU. ^2^ The premix provides per kg of diet: Fe, 120 mg Zn, 100 mg Mn, 20 mg Cu, 1.0 mg I, 0.60 mg Se, and 0.20 mg Co. ^3^ NEmf was referenced from NY/T815-2004 [22]; NFC = 100 − (% CP + % NDF + % EE + % ash) [23].

**Table 2:** Effects of forage-to-concentrate ratio on growth performance and economic benefit in growing yaks.

Items	High Forage	Low Forage	SEM	*p*-Value
Body weight, kg				
Initial	109.61	110.40	2.08	0.857
Day 30	113.56	118.25	2.17	0.295
Day 60	115.86	125.04	2.56	0.072
ADG, g				
Day 0–30	131.67 ^a^	261.55 ^b^	33.20	0.047
Day 30–60	76.56 ^a^	226.37 ^b^	30.88	0.010
Day 0–60	104.11 ^b^	243.96 ^a^	28.74	0.010
Economic benefit				
Feed price (CNY/kg DM)	4.125	5.225		
Feed consumed (kg DM)	60	60		
Feed cost (CNY/yak)	247.5	313.5		
Benefit of LW gain (CNY/yak)	156	366		
Breeding profit (CNY/yak)	−91.5	52.5		
NEB	−0.37	0.17		

ADG = average daily gain; LW = live weight; NEB = net economic benefit. Different lowercase letters within the same row indicate statistically significant differences (*p* < 0.05). *n* = 9.

**Table 3:** Effects of forage-to-concentrate ratio on serum biochemical parameters in growing yaks.

Items	High Forage	Low Forage	SEM	*p*-Value	Reference Intervals
Early experimental stage					
TP, g/L	56.79 ^a^	60.85 ^b^	1.57	0.021	55–76, g/L [25]
ALB, g/L	31.42	32.37	0.71	0.200	27–39, g/L [25]
GLO, g/L	25.37 ^a^	28.48 ^b^	1.36	0.039	28–37, g/L [25]
GLU, mmol/L	4.86	5.10	0.19	0.224	4.32–5.81, mmol/L [26]
BUN, mmol/L	3.91	4.35	0.63	0.488	1.8–7.1, mmol/L [26]
TC, mmol/L	2.94	2.91	0.12	0.820	2.1–8.3, mmol/L [26]
TG, mmol/L	0.51	0.51	0.00	0.758	0.1–0.9, mmol/L [26]
FFA, μmol/L	87.50	86.88	3.18	0.847	-
LDL-C, mmol/L	1.37	1.32	0.10	0.617	-
HDL-C, mmol/L	1.32	1.37	0.07	0.505	-
Late experimental stage					
TP, g/L	59.04 ^a^	61.86 ^b^	0.92	0.008	55–76, g/L [25]
ALB, g/L	32.24	32.45	0.55	0.699	27–39, g/L [25]
GLO, g/L	26.80 ^a^	29.41 ^b^	0.81	0.006	28–37, g/L [25]
GLU, mmol/L	4.75	4.44	0.19	0.126	4.32–5.81, mmol/L [26]
BUN, mmol/L	3.91	3.84	0.57	0.893	1.8–7.1, mmol/L [26]
TC, mmol/L	3.26	3.11	0.22	0.491	2.1–8.3, mmol/L [26]
TG, mmol/L	0.51	0.51	0.00	0.554	0.1–0.9, mmol/L [26]
FFA, μmol/L	91.38	91.38	2.84	1.000	-
LDL-C, mmol/L	1.54	1.42	0.14	0.412	-
HDL-C, mmol/L	1.43	1.38	0.06	0.481	-

TP = total protein; ALB = albumin; GLO = globulin; GLU = glucose; BUN = blood urea nitrogen; TC = total cholesterol; TG = triglycerides; FFA = free fatty acids; LDL-C = low-density lipoprotein cholesterol; HDL-C = high-density lipoprotein cholesterol. Different lowercase letters within the same row indicate statistically significant differences (*p* < 0.05). *n* = 9.

**Table 4:** Effects of forage-to-concentrate ratio on serum hormones in growing yaks.

Items	High Forage	Low Forage	SEM	*p*-Value
Early experimental stage				
T4, pmol/L	1338.58	1338.61	54.04	1.00
T3, pmol/L	94.09	96.45	4.20	0.58
GH, μg/L	42.75 ^a^	47.23 ^b^	1.44	0.01
LEP, μg/L	3.96	4.23	0.15	0.10
INS, mIU/L	45.72	46.34	1.28	0.64
IGF-1, μg/L	32.91	33.60	1.07	0.53
Late experimental stage				
T4, pmol/L	1355.75	1310.07	63.97	0.49
T3, pmol/L	95.46	96.36	3.41	0.80
GH, μg/L	41.95 ^a^	46.23 ^b^	1.38	0.01
LEP, μg/L	3.90	4.12	0.14	0.16
INS, mIU/L	46.97	43.96	1.82	0.12
IGF-1, μg/L	34.45	34.18	1.19	0.82

T4 = thyroxine; T3 = triiodothyronine; GH = growth hormone; LEP = leptin; INS = insulin; IGF-1 = insulin-like growth factor-1. Different lowercase letters within the same row indicate statistically significant differences (*p* < 0.05). *n* = 9.

**Table 5:** Effects of forage-to-concentrate ratio on serum antioxidant capacity in growing yaks.

Items	High Forage	Low Forage	SEM	*p*-Value
Early experimental stage				
SOD, U/mL	5.54	6.17	0.68	0.367
MDA, nmol/mL	0.26	0.19	0.04	0.056
CAT, U/mL	10.48	15.25	3.26	0.163
T-AOC, μmol/mL	0.55	0.53	0.05	0.643
GSH-Px, U/mL	0.34	0.35	0.01	0.655
Late experimental stage				
SOD, U/mL	5.52	5.29	0.80	0.779
MDA, nmol/mL	0.22	0.21	0.01	0.324
CAT, U/mL	9.20	10.59	3.40	0.688
T-AOC, μmol/mL	0.58 ^b^	0.47 ^a^	0.05	0.040
GSH-Px, U/mL	0.37	0.38	0.02	0.637

SOD = superoxide dismutase; MDA = malondialdehyde; CAT = catalase; T-AOC = total antioxidant capacity; GSH-Px = glutathione peroxidase. Different lowercase letters within the same row indicate statistically significant differences (*p* < 0.05). *n* = 9.

## Data Availability

The original contributions presented in this study are included in the article. Further inquiries can be directed to the corresponding author.
